# Catastrophic Floods in Rio Grande do Sul, Brazil: The Need for Public Health Responses to Potential Infectious Disease Outbreaks

**DOI:** 10.1590/0037-8682-0162-2024

**Published:** 2024-05-27

**Authors:** Paulo Ricardo Martins-Filho, Julio Croda, Adriano Antunes de Souza Araújo, Dalmo Correia, Lucindo José Quintans-Júnior

**Affiliations:** 1 Universidade Federal de Sergipe, Laboratório de Patologia Investigativa, Aracaju, SE, Brasil.; 2 Universidade Federal do Mato Grosso do Sul, Departamento de Medicina, Campo Grande, MS, Brasil.; 3 Fundação Oswaldo Cruz, Campo Grande, MS, Brasil.; 4 Yale School of Public Health, Department of Epidemiology of Microbial Diseases, New Haven, CT, USA.; 5 Universidade Federal de Sergipe, Departamento de Farmácia, São Cristóvão, SE, Brasil.; 6 Universidade Federal de Sergipe, Departamento de Medicina, Aracaju, SE, Brasil.; 7 Universidade Federal de Sergipe, Laboratório de Neurociências e Ensaios Farmacológicos, São Cristóvão, SE, Brasil.

The recent floods that have devastated Rio Grande do Sul State in Brazil since late April represent a crisis of historic proportion, affecting approximately 90% of the municipalities. This extreme event, a direct result of human-induced climate change, was driven by a combination of diverse atmospheric factors, including a persistent heatwave from the Central-West and Southeast regions, a humidity corridor from the Amazon, and strong wind currents, leading to unprecedented precipitation and dramatic increases in water levels across the state’s water systems. The entire neighborhood and municipalities were literally underwater, affecting over 2 million people, with 149 deaths and 108 missing people as of May 15, 2024^1^. Beyond the incalculable environmental, social, and economic catastrophes, the risk of infectious disease outbreaks associated with these floods is imminent.

## ● Economic and Human Costs of Inadequate Flood Preparedness

The floods in Rio Grande do Sul are a stark reminder of the escalating threats posed by climate-induced disasters, prompting an urgent need to revisit our national disaster management strategies. According to the Brazilian Institute of Geography and Statistics (IBGE) and National Center for Monitoring and Early Warning of Natural Disasters (Cemaden), more than 8 million Brazilians live in areas at risk of landslides, floods, and other climatic disasters. Over 870 municipalities are identified as risk zones, with approximately 18% of the population in these areas belonging to the most vulnerable age groups, including children under 5 years of age and individuals over 60 years old. In Rio Grande do Sul, more than 270,000 people live in risk zones^2^.

Statistics from the Integrated Disaster Information System (S2iD) of the Ministry of Regional Development revealed a worrying increase in fatalities linked to excessive rainfall. From 2019 to mid-2022, 1,260 deaths were registered, accounting for 71.8% of all disaster-related deaths recorded over the last 10 years^3^. This sharp increase in mortality underscores the escalating effect of extreme weather conditions. Moreover, a significant funding gap exists between crisis management and prevention. From 2013 to 2023, the federal government spent more than R$ 13.3 billion on managing crises resulting from natural disasters, nearly triple the R$ 4.9 billion allocated to prevention measures^4^. This disparity underscores the need for a fundamentally reactive approach for strategic and long-term disaster preparedness. 

The chronic underfunding of preventive measures is alarming, especially given the increasing frequency and severity of floods, which not only claim lives but also trigger widespread socioeconomic disruptions. This financial strategy perpetuates a cycle of unpreparedness that heightens population vulnerability and imposes recurrent economic burdens with each disaster. These findings underscore the urgent need for a strategic shift towards more proactive disaster management. Investing in robust flood prevention and preparedness programs can substantially reduce both the direct human impact and subsequent financial burden of emergency response and recovery. Effective strategies should include enhancing infrastructure resilience, improving early warning systems, and implementing community-based education and training programs to prepare and protect vulnerable populations.

The catastrophic floods in Rio Grande do Sul underscore the critical need for improved preparedness and preventive measures to protect against the increasing risks associated with climate change and extreme weather events. Policymakers must regard these statistics as decisive calls for action, compelling a thorough reassessment and strengthening of the nation's disaster risk management strategies.

## ● Public Health Implications and Potential Infectious Disease Outbreaks

Floodwaters, often contaminated by fecal matter and rodent urine, pose severe health risks by facilitating the spread of diseases such as hepatitis A, typhoid fever, and leptospirosis^5^ ^-^ ^7^. The compromise of sanitary infrastructure during these disasters also leads to contamination of water and food sources, increasing the likelihood of disease and diarrhea outbreaks, particularly affecting older individuals, those with severe dehydration, and people of lower socioeconomic status^8^ ^-^ ^10^. Additionally, floods increase exposure to *Clostridium tetani* spores, which may penetrate the body through wounds or cuts upon contact with contaminated objects and debris^11^. Concurrently, the ecological conditions provided by these climatic anomalies can favor the proliferation of mosquitoes; however, epidemiological studies documenting the impact of flood events on the incidence of dengue and malaria in affected areas are scarce^12^ ^,^ ^13^.

Historical data from Santa Catarina between 2000 and 2016 showed a marked increase in leptospirosis cases following floods, especially in urban settings^14^. The 1996 leptospirosis outbreak in Brazil was a critical case study in which the incidence rates doubled in the flood-prone areas of Rio de Janeiro, demonstrating how floods can significantly amplify disease transmission^15^. In addition, a national ecological study conducted by Gracie et al.^16^, which analyzed the occurrence of leptospirosis and its association with flooding events from 2003 to 2013, found that the risk of leptospirosis was higher in municipalities that experienced floods, regardless of population size. This study also found that as the total number of flooding events in municipalities increased, the incidence of leptospirosis increased. These findings underscore the significant role that natural phenomena play in disease outbreaks and highlight the potential for an increase in the incidence of leptospirosis driven by climate change. As of May 22, 2024, the State Health Department in Rio Grande do Sul has confirmed two fatalities and 29 cases of leptospirosis associated with the recent floods (https://saude.rs.gov.br/confirmado-segundo-obito-por-leptospirose-relacionado-as-enchentes-no-rs).

Similarly, the relationship between flood areas and hepatitis A was explored in a study conducted by Silveira et al.^17^. They structured a database of confirmed cases of hepatitis A and flood events in the municipality of Encantado, Rio Grande do Sul, Brazil, between 2012 and 2014. Their findings revealed that 44 cases of hepatitis A were recorded in the three months following floods, representing an almost 300% increase in incidence. These alarming spikes in post-flood disease cases demonstrate the crucial need for vigilant public health surveillance and preventive strategies to mitigate the spread of infection.

The increasing severity of weather patterns, likely driven by climate change, not only amplifies the spread of waterborne infectious diseases^18^ but also contributes to traumatic injuries, chemical exposure, mental health issues, and a rise in cardiovascular and respiratory morbidity and mortality, particularly affecting low-income and older individuals^19^. The increased risk of cardiovascular mortality following floods can be attributed to several interrelated factors. Physiological and psychological stress from loss, displacement, and uncertainty significantly exacerbate pre-existing heart conditions, potentially leading to heart attacks and strokes. Additionally, exposure to mold and other airborne irritants in flooded homes can exacerbate pre-existing respiratory conditions such as asthma or chronic obstructive pulmonary disease. This highlights the urgent need for effective medical management and preventive strategies to safeguard vulnerable populations both during and after natural disasters.

## ● Mitigation and Future Preparedness

Considering these risks, the response of the health system must be immediate and organized^20^. Public health authorities must develop a robust surveillance system that can promptly identify and address signs of disease outbreaks. This strategy should include mass testing in the affected areas and establishing a dynamic epidemiological surveillance infrastructure designed to quickly trace and manage case development, thereby minimizing further morbidity and mortality associated with flood-related disease outbreaks.

Ensuring access to potable water and establishing safe and hygienic shelters are crucial measures for preventing the spread of diseases. Health education campaigns are equally important for informing the population about the risks of contaminated water and essential hygiene practices to reduce the risk of infection. Furthermore, comprehensive vaccination initiatives should be integrated into disaster response plans to effectively manage and mitigate the health impacts of floods^21^. Beyond the immediate response, reconstructing healthcare infrastructure is crucial for restoring essential services and ensuring the capacity of the health system to handle increased demands. This should include strengthening hospitals and clinics in less affected areas to support the most devastated regions.

Amid this crisis, where emergency teams and volunteers play a vital role in rescue efforts, protecting these individuals becomes a critical component of the overall disaster response. Often, on the front lines, these people are exposed to high risk of contamination from floodwater. Thus, providing them with appropriate personal protective equipment and preventive vaccinations is essential not only for their safety but also for maintaining the efficacy of the disaster response. These preventive measures are vital to ensure that they can continue rescue operations without becoming victims of the same adverse health conditions that they are striving to mitigate.

## CONCLUSION

The response strategy to the floods in Rio Grande do Sul, Brazil, must be multifaceted, addressing both immediate public health needs and long-term challenges of recovery and preventing future outbreaks. Collaboration between governments, health authorities, policymakers, and local communities is crucial for overcoming the effects of this catastrophe.
